# Susceptibility of alpha-1 antitrypsin deficiency variants to polymer-blocking therapy

**DOI:** 10.1172/jci.insight.194354

**Published:** 2025-07-08

**Authors:** Riccardo Ronzoni, Ibrahim Aldobiyan, Elena Miranda, Narinder Heyer-Chauhan, Emma L.K. Elliston, Juan Pérez, Annamaria Fra, James A. Irving, David A. Lomas

**Affiliations:** 1 UCL Respiratory, Division of Medicine, and; 2 Institute of Structural and Molecular Biology, University College London, London, United Kingdom.; 3 Department of Biochemistry, College of Science, King Saud University, Riyadh, Saudi Arabia.; 4 Department of Biology and Biotechnologies ‘Charles Darwin’, Sapienza University of Rome, Rome, Italy.; 5 Department of Cellular Biology, Genetics, and Physiology, University of Malaga, Faculty of Science, Malaga, Spain.; 6 Department of Molecular and Translational Medicine, University of Brescia, Brescia, Italy.

**Keywords:** Cell biology, Clinical Research, Cell stress, Genetic diseases

## Abstract

The Z variant (Glu342Lys) causes alpha-1 antitrypsin (AAT) to self-assemble into polymer chains that accumulate within hepatocytes, causing liver disease and exposing a cryptic epitope recognized by the 2C1 monoclonal antibody (mAb). They can be blocked by the small molecule GSK716 (‘716) that stabilizes an intermediate on the polymerization pathway. We have characterized 23 mutants of AAT in a cellular model to establish: (a) their ability to form intracellular polymers, (b) whether polymer formation could be prevented by ‘716, and (c) whether the polymers expose the 2C1 cryptic epitope. Most of the variants, including Mprocida (Leu41Pro), Mherleen (Pro369Leu), Mduarte (Asp256Val), Lfrankfurt (Pro255Thr), Yorzinuovi (Pro391His), Mwurzburg (Pro369Ser), and p.289S accumulated as intracellular polymers. Eleven formed polymers that were resistant to ‘716, including Mprocida, Mmalton (ΔPhe51), Lfrankfurt, Mduarte, S (Glu264Val), Mherleen, and Yorzinuovi. The ‘716 resistant mutants localize to a region of the AAT molecule separate from the binding site of the small molecule and form polymers that are less well recognized by the 2C1 mAb. They are fully recognized by a novel 8A7 mAb that we developed to have a broader specificity. Our data suggest that individuals with these mutations are unlikely to benefit from treatment with ‘716 or its derivatives.

## Abstract 2

The location of pathogenic mutations in alpha1 antitrypsin correlates with susceptibility to a small-molecule polymerisation inhibitor and with the epitopes displayed by the resulting polymers.

## Graphical abstract

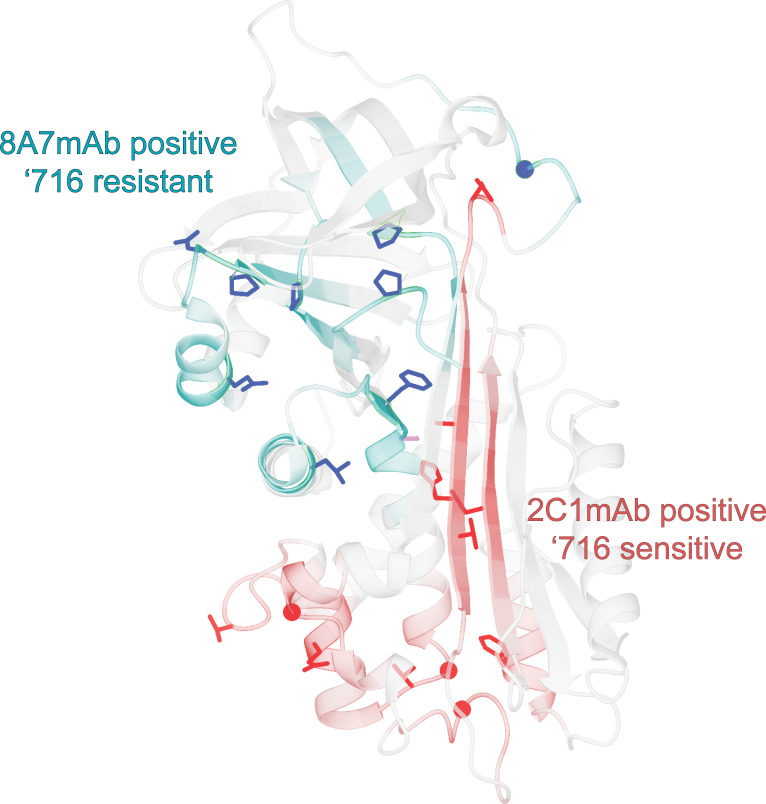

## Introduction

Alpha-1 antitrypsin (AAT) deficiency (AATD) is a monogenic condition resulting from mutations in the *SERPINA1* gene. It is associated with a wide a range of phenotypes and pathological manifestations. Variants characterized by the complete absence of AAT production (often due to nonsense mutations, early stop codons, or gene deletions) are termed Null and are generally described in the literature as Q_0_ alleles (1). Less frequent are the dysfunctional variants that produce normal plasma levels of AAT but with reduced inhibitory activity against neutrophil elastase (2). The most widespread mutations associated with AATD lead to intracellular polymerization of misfolded AAT within hepatocytes. These mutants are classified using the protease inhibitor (Pi) system, based on their electrophoretic mobility in isoelectric focusing. The most common severe deficiency variant is the Z allele (Glu342Lys) that causes the synthesized protein to assemble into polymers with a beads-on-a-string appearance (3). These polymers accumulate within the endoplasmic reticulum (ER) of hepatocytes forming the periodic acid–Schiff^+^ (PAS^+^) inclusions that are characteristic of the condition (4). The misfolding of AAT and accumulation of polymers does not initiate the unfolded protein response within hepatocytes (5–7) but does induce an ER overload response that, in combination with obesity, alcohol excess, and other genetic factors, leads to the development of cirrhosis. The intracellular retention of AAT results in a lack of circulating protein, uncontrolled activity of neutrophil elastase within the lung, and a predisposition to early-onset emphysema (4).

The Z and S (Glu264Val) alleles are universally recognized as the most prevalent disease-associated variants in AATD (8), with Z representing the highest-risk allele, followed by the milder S variant. These pathological alleles have a frequency in non-Finnish European populations of 1.8 × 10^–2^ and 3.7 × 10^–2^, respectively (8). In recent years, an increased focus on screening for this still largely underdiagnosed condition has led to the identification of a growing number of rare AAT variants, many of which are now known to be associated with AATD. With over 2,200 reported cases, the combined frequency of pathological variants such as F (Arg223Cys), I (Arg39Cys), Mmalton (ΔPhe51), and Mduarte (Asp256Val) approaches the prevalence of the Z allele, in regions where screening is more accurate and cohorts are larger (9). The penetrance of AATD varies depending on genotype, with a high but incomplete risk of lung and liver disease in individuals homozygous for Z-AAT (PiZZ) and a lower and environment-related risk in individuals with the SZ and MZ alleles. The effects of rare or null alleles on the penetrance of the phenotype is variable and heterogeneous, and these effects depend on the phenotypes’ heterozygosity with other mild or severe alleles such as S or Z and external factors (10).

In the last 3 decades, various models have been proposed to describe the aberrant protein linkage underlying the pathological polymers of AAT (10). Our recent data, based on purified Z AAT polymers from explanted liver, supports a model in which the C-terminal domain is exchanged sequentially between neighboring AAT molecules of the polymeric chain (11–15). These polymers then condense to form intracellular inclusions (3, 16).

We have developed a mAb, 2C1, that recognizes only the polymeric form of AAT (17). It binds to polymers formed by Z AAT, the antigen used for its development, and to polymers formed by the Siiyama (Ser53Phe), King’s (His334Asp), Pbrescia (Gly225Arg), Baghdad (Ala336Pro), Bologna (Asn186Tyr), and Sydney (Gly192Cys) variants (17–23). These polymers are recognized in cell models of disease and, where this has been assessed, in clinical samples (17, 20, 21, 23). The mAb 2C1 shows a lower affinity for polymers of the Trento variant (Glu75Val) (24). We have also developed a small molecule chaperone (GSK716; ‘716) that blocks polymerization and promotes secretion of monomeric AAT (18, 19). It does this by stabilizing a monomeric, near-native folding intermediate on the polymerization pathway that is favored by the Z variant (18, 19, 25). This molecule is active in vitro, in cells, and in a mouse model of disease, demonstrating that the target AAT conformation is present in all 3 contexts. We report here the characterization of a diverse set of 23 variants of AAT, to establish if they form polymers and whether they are similarly amenable to the polymer-blocking modality of ‘716. Many of the variants have not been fully biochemically characterized; some have been identified in individuals already diagnosed with lung or liver disease associated with AATD, while others have been predicted to be polymerogenic through in silico analysis (26). Assessment of effect of ‘716 has important implications for the generality of the therapeutic prevention of polymerization by monomer stabilization as small molecule polymer blocker therapy is now in clinical trials (27). We also describe the generation of a potentially novel mAb that recognizes all types of polymers, including those formed by the AAT mutants resistant to ‘716.

## Results

### AAT accumulation within the insoluble cellular fraction is observed with multiple variants.

**Figure 1:**
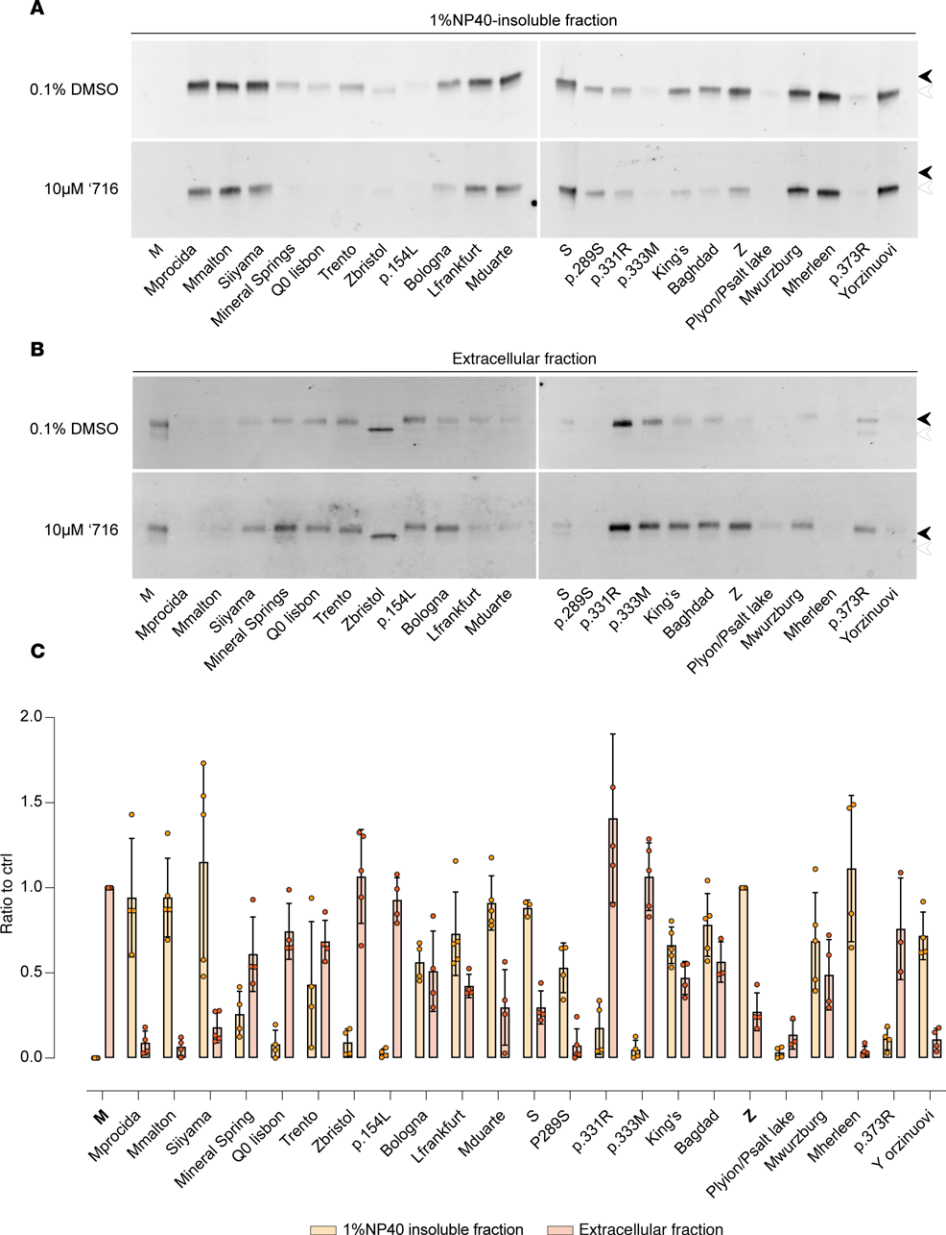
Characterization of AAT mutants and assessment of resistance to the polymerization inhibitor ‘716. (**A** and **B**) Retention within the 1% v/v NP40-insoluble fraction (**A**) and secretion of mutants of AAT (**B**). Cells treated with DMSO (upper panels) or 10 μM ‘716 (lower panels) were analyzed by 4%–12% w/v SDS-PAGE and Western blot after 48 hours following transfection. Black and white arrowheads indicate the mature and immature glycosylated forms of AAT, respectively. (**C**) Densitometric quantification of 1% NP40 insoluble and extracellular fraction of AAT expressed as ratios over the Z and M AAT controls, respectively. The graph shows the mean ± SD (*n* = 3).

Twenty-four naturally occurring variants of AAT, including the WT M and the common severe Z deficiency mutant, were selected from the literature and the gnomAD gene variation database (Table 1) (26). The behavior of these variants was analyzed in the murine hepatoma Hepa 1.6 cell line (28). The AAT protein present in 1% v/v NP40-insoluble cell extract has been shown to be composed exclusively of AAT polymers (19), and this fraction was used to assess the propensity for polymer formation (Figure 1A and Supplemental Data; supplemental material available online with this article; https://doi.org/10.1172/jci.insight.194354DS1). WT M AAT did not form polymers, while variants previously found to do so — Mmalton, Siiyama, Trento, Bologna, S, King’s, Baghdad, and Z AAT (3, 17, 20, 23, 24, 29–31) — accumulated within the insoluble fraction. There was evidence of accumulation for most of the other variants not previously characterized in this way; this was most apparent for Mprocida, Lfrankfurt, Mduarte (also referred as Plowell), p.289S, Mwurzburg, Mherleen, and Yorzinuovi AAT (26, 31–37). For most of these mutants, the degree of accumulation correlated with defective secretion of AAT into the extracellular fraction (Figure 1B); the more severe the accumulation inside the cell, the more severe the secretory deficit (Figure 1C). Plyon/Psalt lake and p.373R (38, 39) showed both little accumulation in the intracellular fraction and poor secretion, suggesting that these mutants undergo degradation rather than polymerization. Zbristol (40) disrupts an NXS/T N-linked-glycosylation motif and so migrated faster in both fractions.

### The small molecule ‘716 inhibits polymerization of many, but not all variants of AAT.

The small molecule ‘716 blocks the polymerization of Z AAT by specifically recognizing and stabilizing a nearly native, monomeric M* intermediate on the polymerization pathway and thereby promoting its secretion (18, 25, 41). The cryptic binding pocket that binds ‘716, with both polar and hydrophobic characters, is formed more readily by the Z variant than WT AAT, which is the basis for ‘716 selectivity toward the mutated form (18). Treatment of the cells with 10 μM ‘716, corresponding to a 20-fold excess over the IC_50_ for Z AAT, revealed different behaviors within the panel of mutants (Figure 1, A and B). Variants such as Trento, Bologna, and Baghdad showed sensitivity to treatment with ‘716, while others were resistant, as evidenced by unchanged levels of 1% v/v NP40-insoluble polymers after treatment and no increase in the quantity of secreted AAT. The resistant variants included Mprocida, Mmalton, Lfrankfurt, Mduarte, S, Mherleen, Yorzinuovi, and p.289S (Figure 1, A and B). Siiyama showed partial resistance to treatment with ‘716.

**Figure 2:**
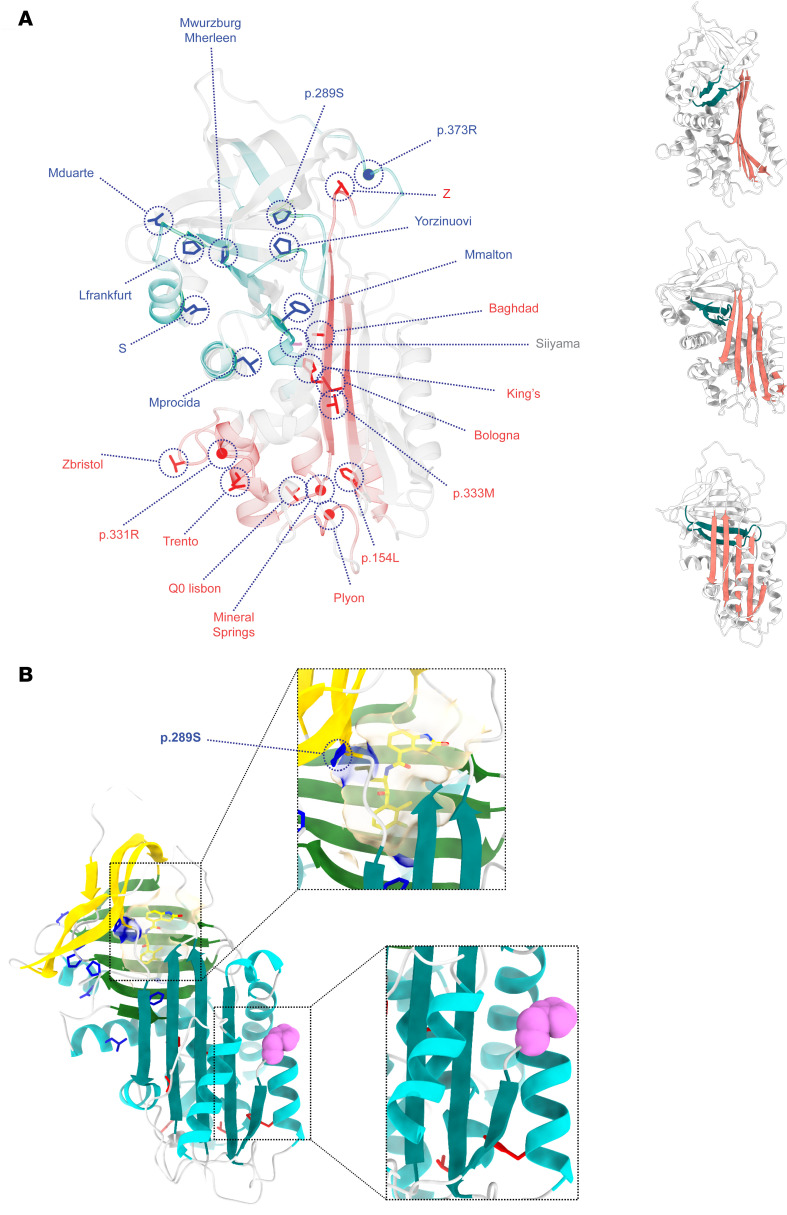
Localization of the mutants assessed in this study on the structure of AAT. (**A**) ‘716-Resistant (blue) and sensitive (red) mutants mapped onto the crystal structure of AAT (PDB 1QLP) (49). The Siiyama mutation is shown in gray. The sensitive and resistant mutations cluster into distinct regions of the protein. Right: AAT in different orientations, with β-sheet A (red) and strands 4B and 5B of the C-terminus (blue) highlighted. (**B**) Top: The ‘716 binding pocket (PDB 7AEL) (18) is affected by the p.289S mutation. Bottom: The 2C1 mAb binding site is located in the vicinity of His139 (pink spheres) (24).

The mutations of the 23 variants were distributed throughout the protein (Table 1). The susceptibility of a mutant to treatment by the small molecule did not correlate with the position of the amino acid substitution within the polypeptide chain. However, there was a striking partition between the mutants when mapped onto the structure of AAT (Figure 2A). Those variants that were resistant to treatment with ‘716 cluster in a distinct lobe of the protein (Figure 2A) that includes the C-terminal β-strands 4B and 5B (Figure 2A); these secondary structural elements are involved in polymer formation by the Z variant (13, 14, 42). In contrast, those variants that were susceptible to compound treatment are located within a cluster of helices in an opposing quadrant of AAT and the central β-sheet A (Figure 2A).

The substitutions associated with ‘716 susceptibility or resistance do not show a consistent physicochemical character. However, the former mostly involve loss of polar contacts, and the latter largely involve residues that form hydrophobic contacts with the protein core, with the introduction of unfavorable packing and likely altered local backbone conformation (Table 2). Among the exceptions, the resistant S and Mduarte mutations disrupt stabilizing polar ionic bonds. The resistant mutations would not be expected to directly affect the binding pocket, and therefore, this resistance likely arises through other effects on the molecule; the outlier, p.289S, affects a proline that contributes to the shape and nonpolar character of the pocket and whose substitution would therefore be more likely to directly affect affinity (Figure 2B). The mutations associated with susceptibility to compound treatment were similarly varied in the effects that they would be predicted to have on the molecule (Table 2).

### AAT variants resistant to ‘716 show mixed dose-response effects.

**Figure 3:**
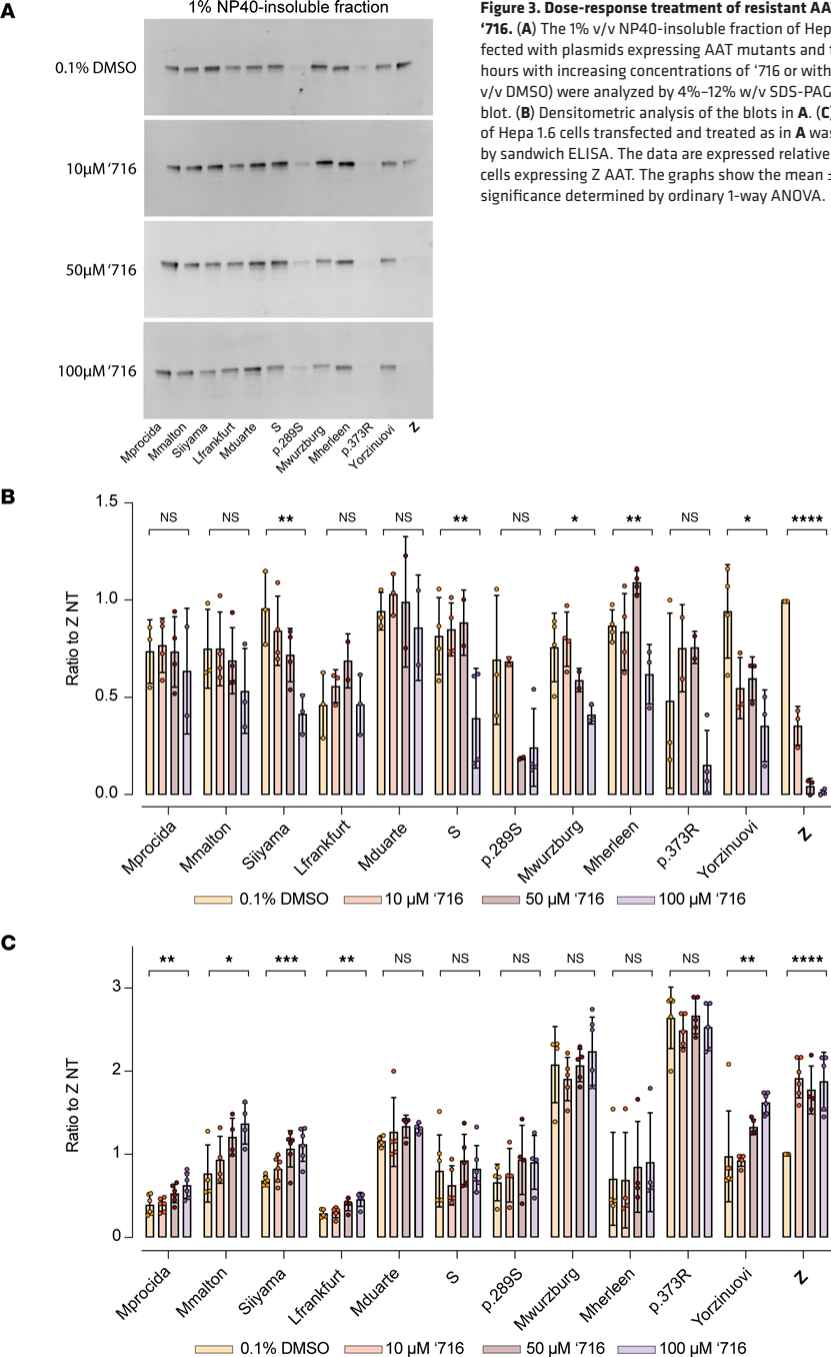
Dose-response treatment of resistant AAT variants with ‘716. (**A**) The 1% v/v NP40-insoluble fraction of Hepa 1.6 cells transfected with plasmids expressing AAT mutants and treated for 48 hours with increasing concentrations of ‘716 or with vehicle (0.1% v/v DMSO) were analyzed by 4%–12% w/v SDS-PAGE and Western blot. (**B**) Densitometric analysis of the blots in **A**. (**C**) Secreted AAT of Hepa 1.6 cells transfected and treated as in **A** was quantified by sandwich ELISA. The data are expressed relative to untreated cells expressing Z AAT. The graphs show the mean ± SD (*n* = 4) with significance determined by ordinary 1-way ANOVA.

Experiments were performed to establish if the resistance to ‘716 could be overcome at higher concentrations of the small molecule. Hepa 1.6 cells transfected with each AAT mutant were incubated with 10, 50, and 100 μM ‘716 (equivalent to 20-, 100- and 200-fold of the Z AAT IC_50_, respectively) for 48 hours. Z AAT was included as a positive control. Higher concentrations of ‘716 resulted in an apparent reduction in intracellular inclusions for Siiyama, S, Mwurzburg, Mheerlen, and Yorzinuovi (Figure 3, A and B). There was also a reduction in the retention of p.289S AAT, although the retention of this mutant was much lower than the others. However, the changes were not statistically significant. There was an increase in the secretion of Mprocida, Mmalton, Siiyama, Lfrankfurt, and Yorzinuovi AAT with increasing concentrations of ‘716 when assessed by ELISA (Figure 3C); thus, only Siiyama and Yorzinuovi showed a recovery by both parameters at high concentrations of the inhibitor.

### Decreased reactivity of the 2C1 mAb against polymers formed by ‘716-resistant AAT variants.

**Figure 4:**
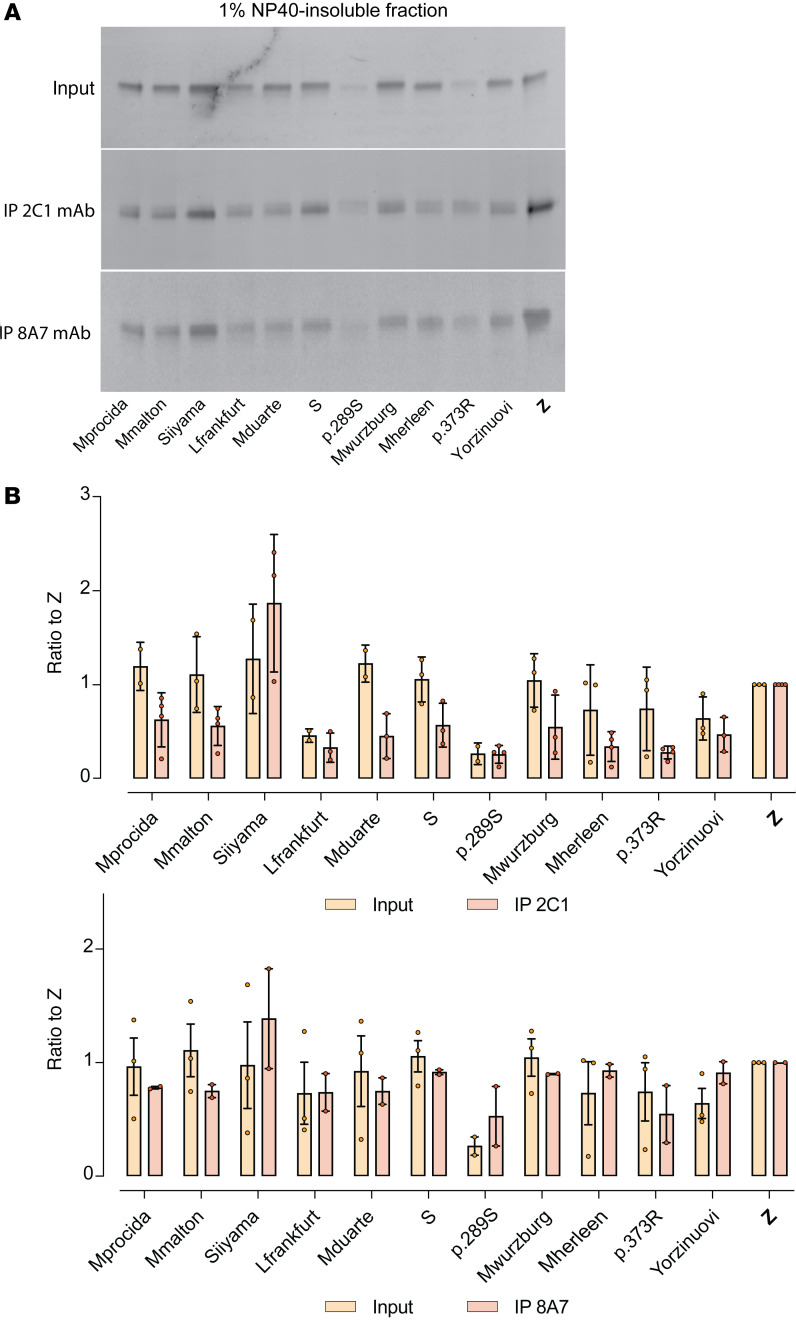
Recognition of ‘716-resistant polymers by the antipolymer 2C1 mAb. (**A**) The 1% v/v NP40 insoluble fractions from cells expressing ‘716-resistant mutants were immunoprecipitated with either the 2C1 mAb (middle panel) or 8A7 mAb (lower panel) and resolved on 4%–12% w/v acrylamide SDS-PAGE. (**B**) Densitometry after Western blot analysis was performed using Image Studio Lite (LI-COR) and are representative of the mean ± SD (*n* = 3). The data for the 2C1 mAb (above) and the 8A7 mAb (below) are expressed relative to cells expressing Z AAT.

The ‘716-resistant mutants were assessed to determine whether the resulting polymers were detected by the 2C1 mAb, which recognizes a cryptic epitope that is exposed by polymers formed by the canonical Z AAT polymerization pathway (17, 25, 43) (Figure 2B). The 1% v/v NP40-insoluble fractions from cells expressing the mutants were immunoprecipitated with the 2C1 mAb and resolved by SDS-PAGE. Cells expressing Z AAT were included as a positive control (Figure 4, A and B). As expected, Z AAT polymers were completely precipitated by the 2C1 mAb, while polymers of the other variants were less well recognized by the antibody (Figure 4, A and B). This was most marked for Mprocida, Mmalton, Mduarte, S, Mwurzburg, Mherleen, p.373R, or the Yorzinuovi mutants. The sites of these mutations are well separated from the 2C1 mAb epitope (24, 42) and, therefore, would not directly interfere with antibody recognition (Figure 2B).

### Development of a novel mAb that recognizes a cryptic epitope with broader antipolymer reactivity.

**Figure 5:**
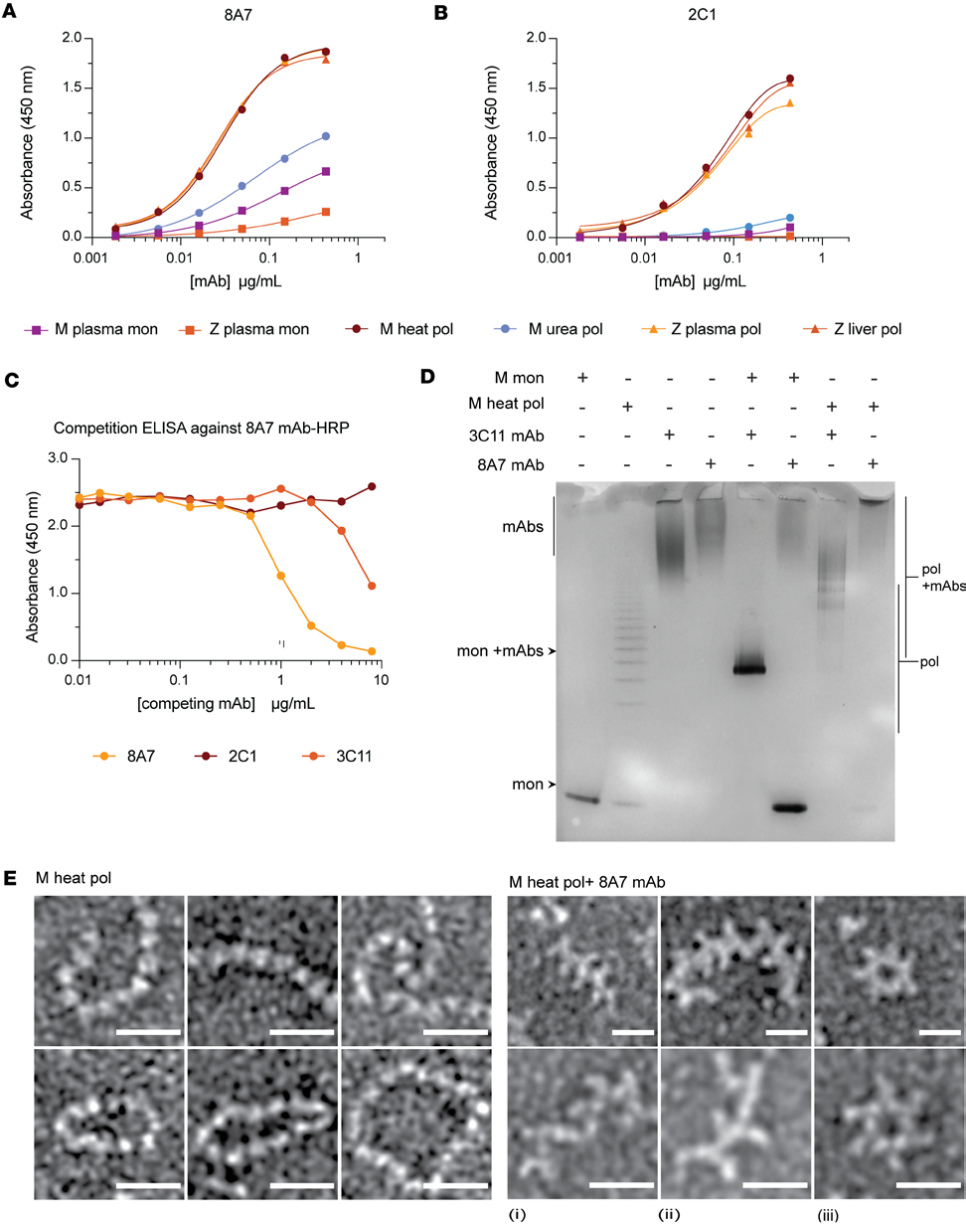
Assessment of the specificity of the novel 8A7 mAb. (**A** and **B**) The 8A7 (**A**) and 2C1 (**B**) mAbs were assessed against different AAT conformers (generated as specified in Methods). (**C**) Competition ELISA between HRP-labeled 8A7 and 2C1 and a nonconformationally selective mAb (3C11), or unlabeled 8A7 as a positive control, against heat-induced M polymers in an antigen ELISA (19, 44, 48). (**D**) Electrophoretic mobility shift assays showing reactivity of the 8A7 mAb against polymeric AAT. (**E**) Negative stain electron microscopy was used to visualize the interaction between plasma M heat-induced polymers made with M AAT from plasma and the 8A7 fragment antigen-binding (Fab) domain. Scale bar: 20 nm. Images were low-pass filtered to 8Å to enhance contrast.

The accumulation of ‘716-resistant mutants of AAT in the 1% v/v NP40-insoluble fraction suggests that they form polymers, even though they are less well recognized by the 2C1 mAb. A new mAb to detect a broader range of AAT polymers was therefore developed. The 8A7 mAb recognized all polymeric forms of AAT by sandwich ELISA, irrespective of their source or means of production (Figure 5). The highest affinity was for heat-induced polymers made from M AAT purified from plasma (Figure 5A) that are comparable with the Z AAT polymers that deposit in liver tissue (17) and also are strongly recognized by the 2C1 mAb (Figure 5B). The high affinity for denaturant-induced polymers by 8A7 mAb contrasts with the low affinity of the 2C1 mAb (Figure 5B) (17). There was little recognition of monomeric AAT by either the 2C1 or 8A7 mAb, and there was no evidence of competition between the 2 mAbs when used to detect heat-induced M polymers, supporting that they bind to different epitopes (Figure 5C). An electrophoretic mobility shift assay (EMSA) similarly confirmed binding of 8A7 to polymeric AAT but not to monomer in contrast to the nonconformation selective 3C11 mAb (44) (Figure 5D). Heat-induced AAT polymers visualized by negative stain electron microscopy (Figure 5E) exhibited readily identifiable protrusions upon incubation with the antibody fragment (Fab) domain of 8A7. Hence, the potentially novel 8A7 mAb is polymer selective but with a broader reactivity than the 2C1 mAb.

### The ‘716-resistant variants produce polymers detectable with the 8A7 mAb.

**Figure 6:**
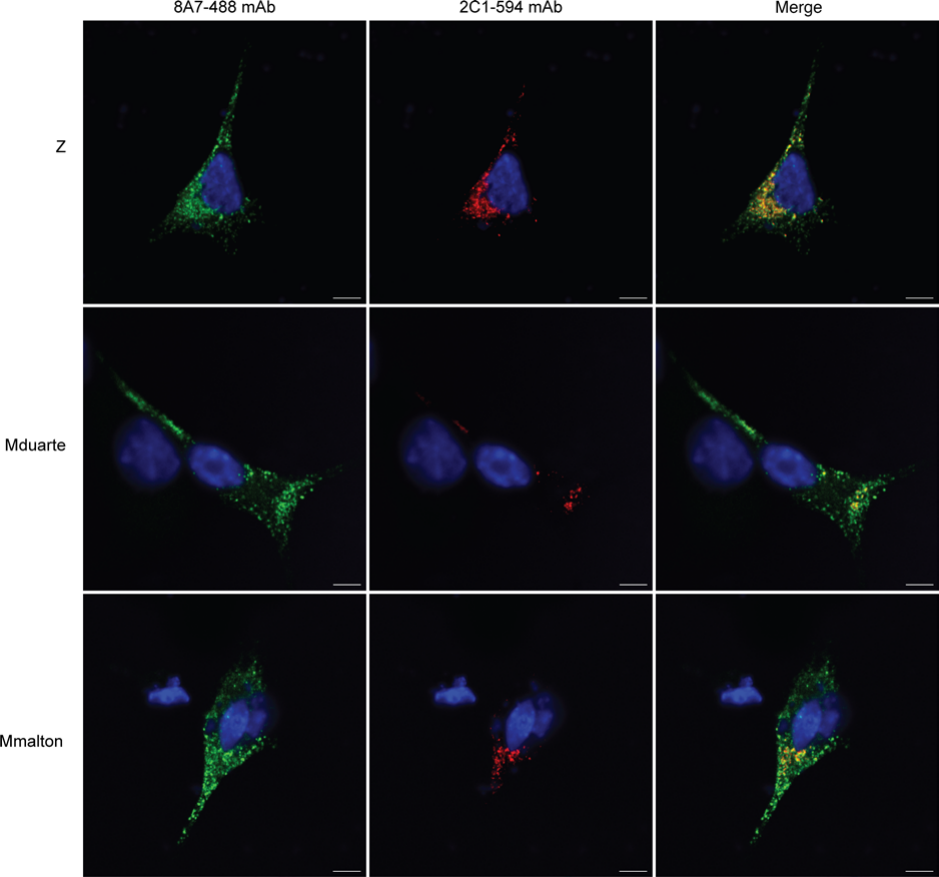
The 8A7 mAb recognizes polymers of AAT variants poorly recognized by the 2C1 mAb. Immunofluorescence of transfected Hepa 1.6 cells stained with the 2C1 mAb directly conjugated with ATTO-594 (red) and the 8A7 mAb directly conjugated with ATTO-488 (green). Scale bar: 10 μm.

The 8A7 mAb was used to determine whether the poor recognition of the ‘716-resistant AAT variants by the 2C1 mAb was due to an alternate polymer form or an absence of polymer formation. In immunoprecipitation experiments, the 8A7 mAb was able to capture these variants in proportion to the amount of the input, indicating that they do accumulate as polymers (Figure 4, A and B). When stained by immunofluorescence with the 8A7 mAb, 2 of the variants, Mduarte and Mmalton AAT, exhibited an extensive punctate distribution consistent with the accumulation of material in inclusion bodies, despite limited recognition by 2C1 mAb (Figure 6).

## Discussion

Over 120 different missense variants of AAT have been described, of which approximately 50 are associated with some degree of deficiency in AAT levels (45). Among the 23 variants that we have analyzed here, the majority form intracellular polymers that can be abrogated by treatment with the polymerization inhibitor ‘716. The biochemical behavior of these variants is in keeping with that described previously for the common severe Z deficiency variant (18, 19). In addition to sensitivity to ‘716, the polymers of these mutants are readily recognized by the 2C1 mAb (17).

A subset of the polymerogenic AAT mutants showed partial or complete resistance to ‘716 treatment. In these cases, there was no rescue of their intracellular accumulation as assessed by reduction in the insoluble fraction and increase in secretion. This was despite increasing the concentration of ‘716 to up to 200 times the IC_50_ determined for Z AAT. For one of these mutations, p.289S, the lack of effect can be readily explained as it affects an amino acid that forms part of the ‘716 binding pocket. Its effect on compound binding is therefore likely to be direct. Individuals with mutations that are resistant to ‘716 are unlikely to benefit from this therapy.

It is notable that resistance to ‘716 treatment corresponded with poor recognition by the 2C1 mAb of material within the insoluble cellular fraction. The 2C1 antibody recognizes a cryptic epitope that is presented by naturally arising Z AAT polymers and is absent in pH and denaturant-induced AAT polymers (46). This suggests that these variants may give rise to a different structural polymorph. Our development of the 8A7 mAb with broader recognition of a range of polymers has allowed us to conclude that these variants do indeed produce polymers but with a different immunorecognition profile. The correspondence between insensitivity to ‘716 and lower recognition by the 2C1 mAb, together with strong binding by the 8A7 mAb, suggests that the combined use of these antibodies could provide a means to predict polymerogenic mutants of AAT that are resistant to ‘716.

A derivative of ‘716, BMN349, is now in clinical trials (27) for individuals who are either PiZZ homozygotes or PiMZ heterozygotes (carrying 1 M- and 1 Z-AAT allele) with metabolic dysfunction–associated steatohepatitis. Our data suggest that there is a wider group of individuals with AAT variants who may respond to therapy with ‘716 or its derivatives.

## Methods

### Sex as a biological variable.

As the work was performed in cell lines, sex was not considered as a biological variable in our studies.

### Cell lines, transfection and characterization of intracellular and extracellular AAT.

The Hepa 1.6 mouse hepatoma cell line (ATCC CRL-1830) was cultured in DMEM. Cells were transfected with plasmids encoding the AAT variants, as described previously (28), and lysed at a concentration of 2.5 × 10^6^ cells/mL in 1% v/v NP-40 buffer (10 mM Tris [pH 7.4], 300 mM NaCl, 1% v/v NP-40) supplemented with protease inhibitors (Roche Ltd.). Cell lysates were then mixed on a rotator for 30 minutes at 4°C. 1% v/v NP-40–soluble and –insoluble fractions were separated by centrifugation at 16,000g for 15 minutes at 4°C. The supernatant (1% v/v NP-40–soluble fraction) was retained; the pellet representing the 1% v/v NP-40–insoluble fraction was then washed twice in 1% v/v NP-40 buffer and mechanically resuspended in an equal volume of lysis buffer and disrupted (max amplitude for 3× 15 seconds) in a chilled cup horn sonicator (Qsonica). The resulting 1% v/v NP-40–soluble and –insoluble intracellular and secreted AAT was assessed by SDS-PAGE and nondenaturing PAGE, immunoblots, ELISA, and EMSA (17, 19, 25, 47).

### Secretion assay.

Sixteen hours before the lysis, cells were washed twice in prewarmed PBS and incubated at 37°C with Opti-MEM (Thermo Fisher Scientific). Cell media were then collected, centrifuged at 300*g* for 5 minutes at 4 °C, and transferred into a clean tube to be subjected to SDS PAGE or immunoprecipitation.

### Immunoprecipitation and Western blot.

Cell lysates or culture media were mixed on a rotator with 1 μg of suitable mAb for 1 hours and recombinant protein G agarose beads (Thermo Fisher Scientific) overnight at 4°C. Beads were then collected by centrifugation (15,000*g* at 4ºC), washed 3 times with 1% v/v NP-40 buffer, washed once with 10 mM Tris (pH 7.4), and eluted in loading buffer (New England Biolabs) in reducing conditions at 93°C for 5 minutes. The eluate was then resolved on 4%–12% w/v acrylamide SDS-PAGE (Bio-Rad).

Following SDS-PAGE, samples were transferred to LF-PVDF membrane (MilliporeSigma), saturated in 5% w/v low-fat milk (New England Biolabs) in PBS-0.1% v/v Tween, probed with polyclonal rabbit anti-AAT (Dako, Agilent; A0012), and detected with goat anti–rabbit antibody conjugated to Alexa Fluor 488 (Thermo Fisher Scientific, A32731). Western blot images were acquired with the iBright1500 (Thermo Fisher Scientific) and analyzed with IMAGE STUDIOLITE software (LI-COR Biosciences).

### Immunofluorescence analysis.

Hepa 1.6 cells were seeded onto 2 cm^2^ coverslips (MilliporeSigma), transfected with vectors encoding AAT variants, fixed after 48 hours with ice-cold methanol, permeabilized with 0.1% v/v Triton X-100, and immunodecorated overnight at 4ºC with (a) anti-AAT polymer 2C1 mAb (17) (1.5 μg/mL) conjugated with Atto-488 (ATTO-TEC GmbH) or (b) anti-AAT polymer 8A7 mAb (1.5 μg/mL) conjugated with Atto-594 (ATTO-TEC GmbH). Nuclei were stained with Hoechst (Thermo Fisher Scientific), and the cells were analyzed on a Zeiss Airyscan 880 confocal microscope with a 63× objective (1.4 oil).

### Production of the 8A7 anti-AAT polymer mAb.

Z AAT monomers and polymers, and M AAT monomers were purified from human plasma as described previously (24, 43). Artificial polymers of WT M AAT were produced over 48 hours through destabilization by: (a) heating M AAT at 55°C, (b) pH 4.5 at 25°C, or (c) in the presence of 4 M urea at 25°C (46). After dialysis into 20 mM Tris (pH 8.0), the resulting polymers were separated from residual monomers by anion exchange chromatography (13). Hybridomas were generated from the splenocytes of mice immunized with these polymers and characterized by ELISA as described previously (17). mAbs were purified from the culture media using a HiTrap Protein G column (Cytiva) (48) and stored in phosphate-buffered saline (PBS) with 0.02% (w/v) sodium azide. Fab fragments of 8A7 were generated by limited proteolysis using ficin according to the manufacturer’s instructions (Thermo Fisher Scientific) with the subsequent addition of 1 mM E-64 inhibitor. Liver polymers were extracted as previously described (13).

### Negative stain electron microscopy of M AAT polymer in complex with 8A7 monoclonal Fab.

Heat-induced plasma M AAT polymers were incubated with a 3-fold molar excess of 8A7 Fab for 3 hours at room temperature, and the sample repurified by gel filtration chromatography on a Superdex 200 Increase 10/30 GL column (Cytiva) in EM buffer (5 mM EDTA, 50 mM NaCl, and 50 mM Tris [pH 7.4]). In total, 3 μL of sample were negatively stained in 2% w/v uranyl acetate on 300 mesh copper grids (Agar Scientific) at ~0.05 mg/mL, as described previously (13). Single-frame high-contrast micrographs of the samples were recorded using an FEI Tecnai T12 microscope operating at 120 kV from a LaB6 emitter; data were recorded on a Gatan CCD MultiScan camera at an effective magnification of ×67,000 and a defocus range of 2–3 μm.

### Statistics.

All statistical analyses was performed with GraphPad Prism 10.4.1. The significance of the differences related to the intracellular fraction 1% NP40-insoluble and the extracellular fraction (cell media) of cells treated with the maximum amount of ‘716 (100 μM) or with the vehicle (0.1% v/v DMSO). The data shown in Figure 3 were evaluated using ordinary 1-way ANOVA on 4 independent replicates.

### Study approval.

As the work was performed in cell lines, study approval was not necessary.

### Data availability.

The data generated in this study are provided in the main manuscript. Values for all data points in graphs are reported in the Supporting Data Values file.

## Author contributions

RR, JAI, and DAL designed the study. RR, IA, EM, NHC, ELKE, JP, AF, and JAI undertook the experiments and procedures. RR wrote the first draft of the article, which was revised by JAI and DAL and then by all the authors. All the authors approved the final version of the manuscript.

## Supplementary Material

Supplemental data

Unedited blot and gel images

Supporting data values

## Figures and Tables

**Table 1:**
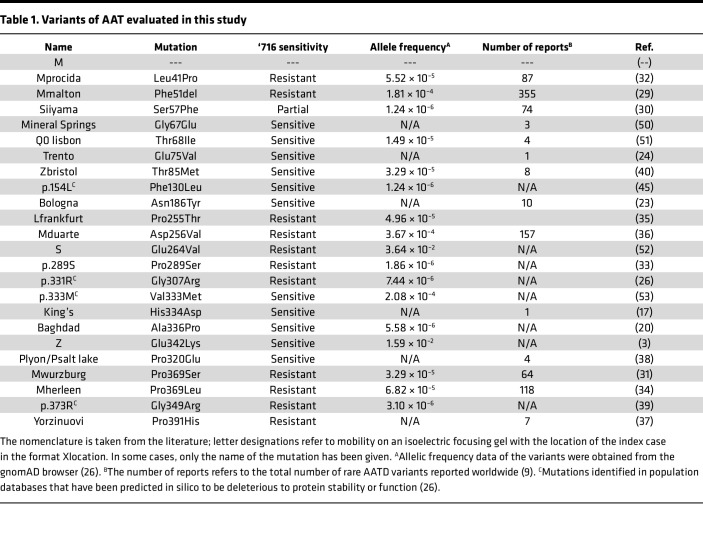
Variants of AAT evaluated in this study

**Table 2:**
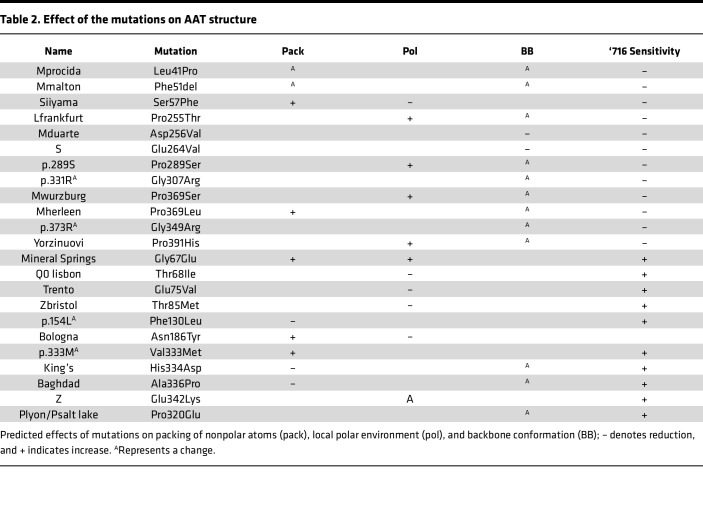
Effect of the mutations on AAT structure

## Version 1 07/08/2025

In-Press Preview

## Version 2 08/08/2025

Electronic publication

## References

[1] Luisetti M, Seersholm N (2004). Alpha1-antitrypsin deficiency. 1: epidemiology of alpha1-antitrypsin deficiency. Thorax.

[2] Carrell RW, Lomas DA (2002). Alpha1-antitrypsin deficiency--a model for conformational diseases. N Engl J Med.

[3] Lomas DA (1992). The mechanism of Z alpha 1-antitrypsin accumulation in the liver. Nature.

[4] Strnad P (2020). Alpha_1_-antitrypsin deficiency. N Engl J Med.

[5] Ordóñez A (2013). Endoplasmic reticulum polymers impair luminal protein mobility and sensitize to cellular stress in alpha1-antitrypsin deficiency. Hepatology.

[6] Graham KS (1990). Accumulation of the insoluble PiZ variant of human alpha 1-antitrypsin within the hepatic endoplasmic reticulum does not elevate the steady-state level of grp78/BiP. J Biol Chem.

[7] Hidvegi T (2005). Accumulation of mutant alpha1-antitrypsin Z in the endoplasmic reticulum activates caspases-4 and -12, NFkappaB, and BAP31 but not the unfolded protein response. J Biol Chem.

[8] Ghosh AJ, Hobbs BD (2022). Recent advancements in understanding the genetic involvement of alpha-1 antitrypsin deficiency associated lung disease: a look at future precision medicine approaches. Expert Rev Respir Med.

[9] Ferrarotti I (2024). Rare variants in alpha 1 antitrypsin deficiency: a systematic literature review. Orphanet J Rare Dis.

[10] Greene CM (2016). Otitis media. Nat Rev Dis Primers.

[11] Yamasaki M (2011). Molecular basis of α1-antitrypsin deficiency revealed by the structure of a domain-swapped trimer. EMBO Rep.

[12] Yamasaki M (2008). Crystal structure of a stable dimer reveals the molecular basis of serpin polymerization. Nature.

[13] Faull SV (2020). The structural basis for Z α_1_-antitrypsin polymerization in the liver. Sci Adv.

[14] Vickers S (2025). Top-down ion mobility mass spectrometry reveals a disease associated conformational ensemble of alpha-1-antitrypsin. J Am Chem Soc.

[15] Lowen SM (2025). High-resolution characterization of ex vivo AAT polymers by solution-state NMR spectroscopy. Sci Adv.

[16] Chambers JE (2022). Z-α_1_-antitrypsin polymers impose molecular filtration in the endoplasmic reticulum after undergoing phase transition to a solid state. Sci Adv.

[17] Miranda E (2010). A novel monoclonal antibody to characterize pathogenic polymers in liver disease associated with alpha1-antitrypsin deficiency. Hepatology.

[18] Lomas DA (2021). Development of a small molecule that corrects misfolding and increases secretion of Z α_1_ -antitrypsin. EMBO Mol Med.

[19] Ronzoni R (2021). The molecular species responsible for α_1_ -antitrypsin deficiency are suppressed by a small molecule chaperone. FEBS J.

[20] Haq I (2016). Deficiency mutations of alpha-1 antitrypsin. Effects on folding, function, and polymerization. Am J Respir Cell Mol Biol.

[21] Kamuda K (2024). A novel pathological mutant reveals the role of torsional flexibility in the serpin breach in adoption of an aggregation-prone intermediate. FEBS J.

[22] Medicina D (2009). Molecular characterization of the new defective P(brescia) alpha1-antitrypsin allele. Hum Mutat.

[23] Ronzoni R (2021). The importance of N186 in the alpha-1-antitrypsin shutter region is revealed by the novel bologna deficiency variant. Int J Mol Sci.

[24] Miranda E (2017). The pathological Trento variant of alpha-1-antitrypsin (E75V) shows nonclassical behaviour during polymerization. FEBS J.

[25] Jagger AM (2020). High-resolution ex vivo NMR spectroscopy of human Z α_1_-antitrypsin. Nat Commun.

[26] Giacopuzzi E (2018). Real-world clinical applicability of pathogenicity predictors assessed on SERPINA1 mutations in alpha-1-antitrypsin deficiency. Hum Mutat.

[27] https://clinicaltrials.biomarin.com/clinical-trial/study-of-bmn-349-single-dose-in-pizz-and-pimz-adult-participants/.

[28] Ronzoni R (2016). Aberrant disulphide bonding contributes to the ER retention of alpha1-antitrypsin deficiency variants. Hum Mol Genet.

[29] Lomas DA (1995). Alpha 1-antitrypsin Mmalton (Phe52-deleted) forms loop-sheet polymers in vivo. Evidence for the C sheet mechanism of polymerization. J Biol Chem.

[30] Lomas DA (1993). Alpha 1-antitrypsin Siiyama (Ser53-->Phe). Further evidence for intracellular loop-sheet polymerization. J Biol Chem.

[31] Poller W (1999). Molecular characterisation of the defective alpha 1-antitrypsin alleles PI Mwurzburg (Pro369Ser), Mheerlen (Pro369Leu), and Q0lisbon (Thr68Ile). Eur J Hum Genet.

[32] Takahashi H, Crystal RG (1990). Alpha 1-antitrypsin Null(isola di procida): an alpha 1-antitrypsin deficiency allele caused by deletion of all alpha 1-antitrypsin coding exons. Am J Hum Genet.

[33] Kueppers F (2021). Clinical presentations of four patients with rare alpha 1 antitrypsin variants identified in a single US center. Respir Med Case Rep.

[34] Hofker MH (1989). A pro----leu substitution in codon 369 of the alpha-1-antitrypsin deficiency variant PI MHeerlen. Hum Genet.

[35] Quinn M, Turner AM (2020). Modernising case finding for α_1_-antitrypsin deficiency by DNA sequencing of COPD patients. Eur Respir J.

[36] Lieberman J (1976). A new deficient variant of alpha1-antitrypsin (MDUARTE). Inability to detect the heterozygous state by antitrypsin phenotyping. Am Rev Respir Dis.

[37] Fra AM (2012). Three new alpha1-antitrypsin deficiency variants help to define a C-terminal region regulating conformational change and polymerization. PLoS One.

[38] Bornhorst JA (2007). Genotypes and serum concentrations of human alpha-1-antitrypsin “P” protein variants in a clinical population. J Clin Pathol.

[39] Laffranchi M (2019). Characterisation of a type II functionally-deficient variant of alpha-1-antitrypsin discovered in the general population. PLoS One.

[40] Bates K (2013). A patient with the rare alpha-1-antitrypsin variant (Z)bristol in compound heterozygosity with the Z mutation. Ann Clin Biochem.

[41] Irving JA (2015). An antibody raised against a pathogenic serpin variant induces mutant-like behaviour in the wild-type protein. Biochem J.

[42] Lowen SM (2025). High-resolution characterisation of ex vivo AAT polymers by solution-state NMR spectroscopy. Sci Adv.

[43] Irving JA (2014). Altered native stability is the dominant basis for susceptibility of α1-antitrypsin mutants to polymerization. Biochem J.

[44] Tan L (2014). Circulating polymers in α1-antitrypsin deficiency. Eur Respir J.

[45] Silva D (2016). Alpha-1-antitrypsin (SERPINA1) mutation spectrum: three novel variants and haplotype characterization of rare deficiency alleles identified in Portugal. Respir Med.

[46] Ekeowa UI (2010). Defining the mechanism of polymerization in the serpinopathies. Proc Natl Acad Sci U S A.

[47] Belorgey D (2011). Characterisation of serpin polymers in vitro and in vivo. Methods.

[48] Ordóñez A (2015). A single-chain variable fragment intrabody prevents intracellular polymerization of Z α1-antitrypsin while allowing its antiproteinase activity. FASEB J.

[49] Elliott PR (1998). Wild-type alpha 1-antitrypsin is in the canonical inhibitory conformation. J Mol Biol.

[50] Curiel DT (1990). Molecular basis of alpha 1-antitrypsin deficiency and emphysema associated with the alpha 1-antitrypsin Mmineral springs allele. Mol Cell Biol.

[51] Faber JP (1994). Identification and DNA sequence analysis of 15 new alpha 1-antitrypsin variants, including two PI*Q0 alleles and one deficient PI*M allele. Am J Hum Genet.

[52] Curiel DT (1989). Serum alpha 1-antitrypsin deficiency associated with the common S-type (Glu264----Val) mutation results from intracellular degradation of alpha 1-antitrypsin prior to secretion. J Biol Chem.

[53] Kueppers F (2019). Protein modeling to assess the pathogenicity of rare variants of SERPINA1 in patients suspected of having alpha 1 antitrypsin deficiency. BMC Med Genet.

