# Supplementary material for: Susceptibility of alpha-1 antitrypsin deficiency variants to polymer-blocking therapy
Source: JCI Insight. 2025 Jul 8;10(15):e194354. doi: 10.1172/jci.insight.194354 (PMC12333946; doi:10.1172/jci.insight.194354)
Supplement: Supplemental data [file jciinsight-10-194354-s139.pdf]

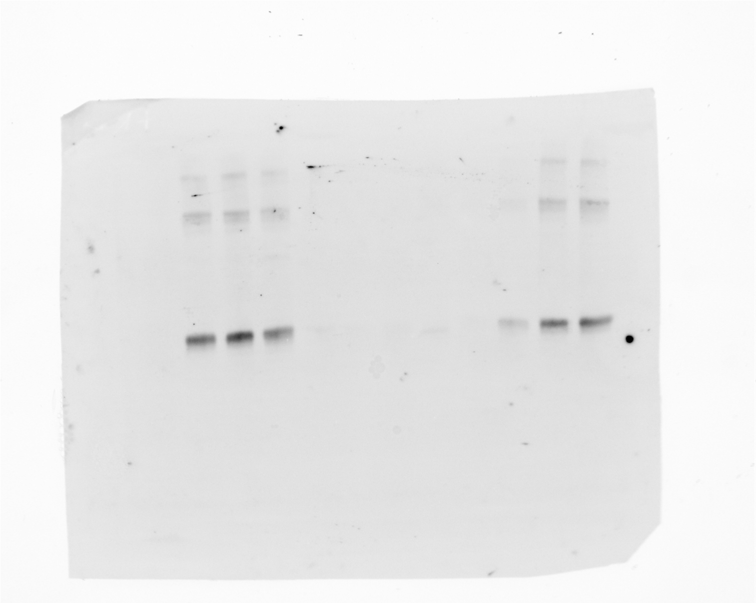

Full unedited gel for Figure 1A bottom left

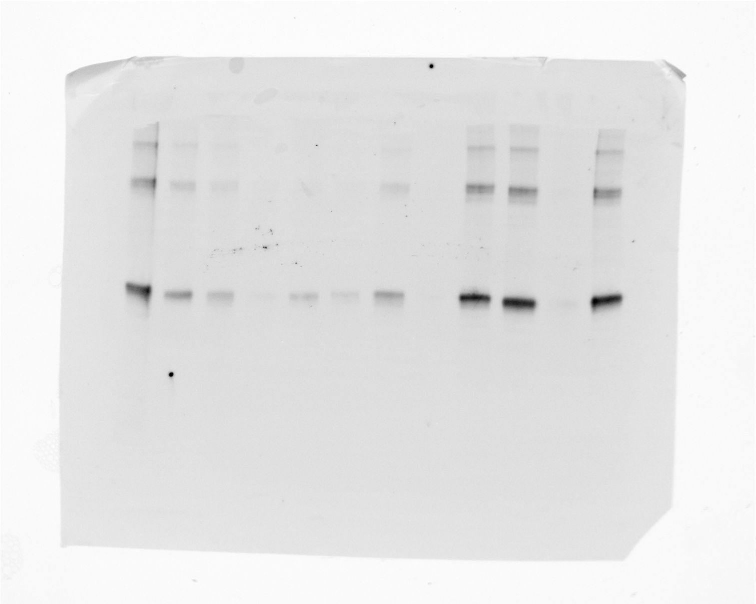

Full unedited gel for Figure 1A bottom right

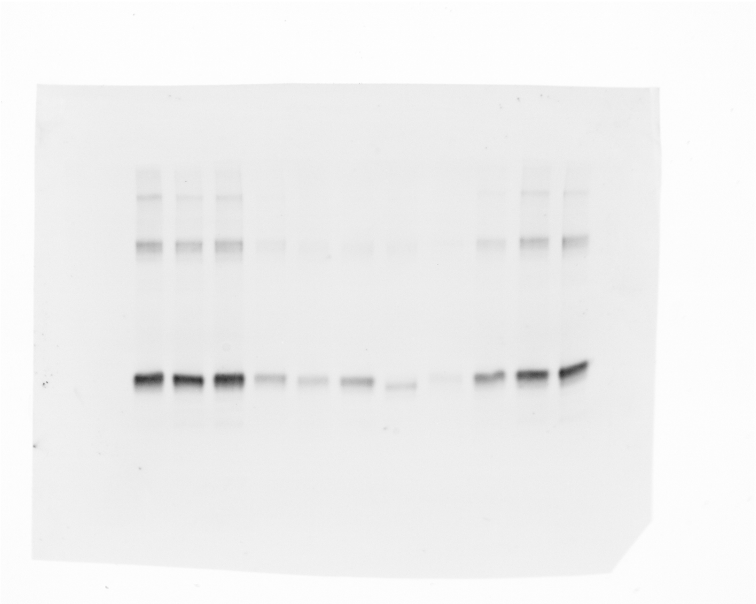

Full unedited gel for Figure 1A top left

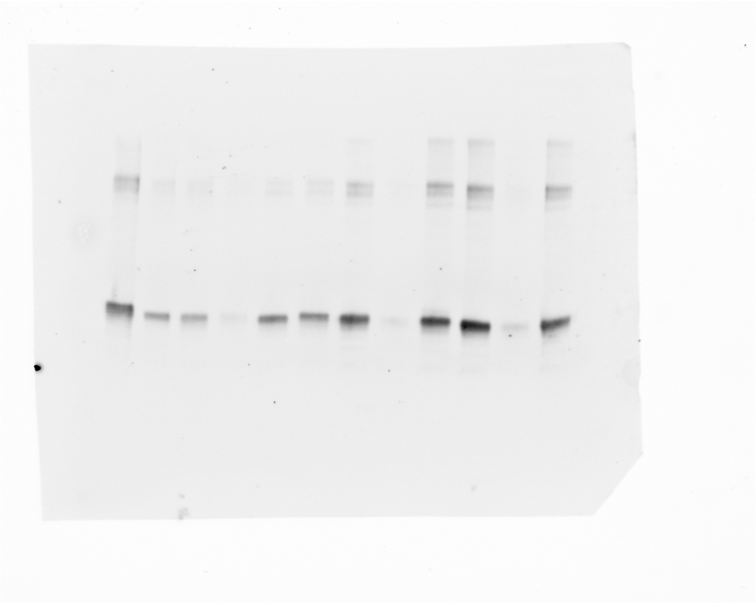

Full unedited gel for Figure 1A top right

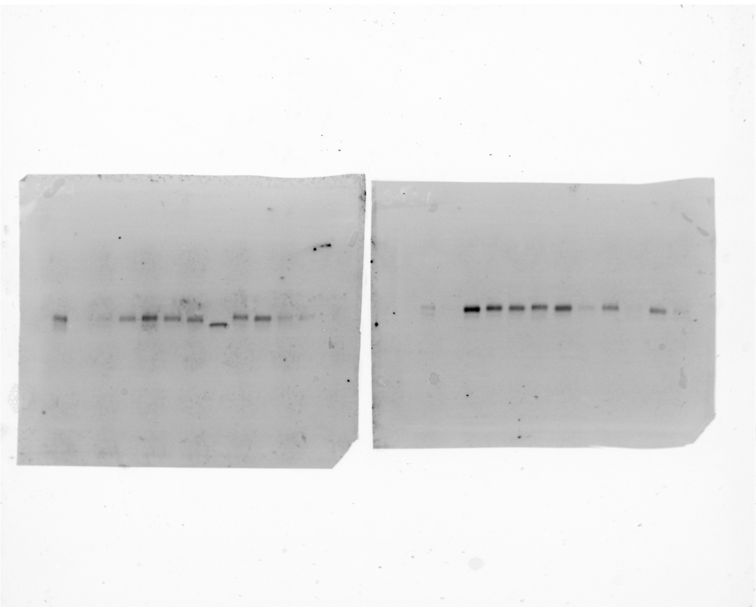

Full unedited gel for Figure 1B bottom

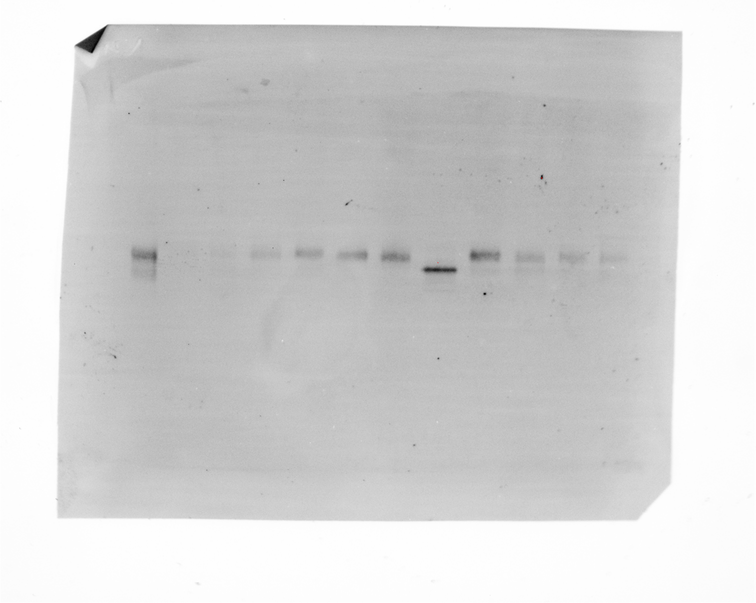

Full unedited gel for Figure 1B top left

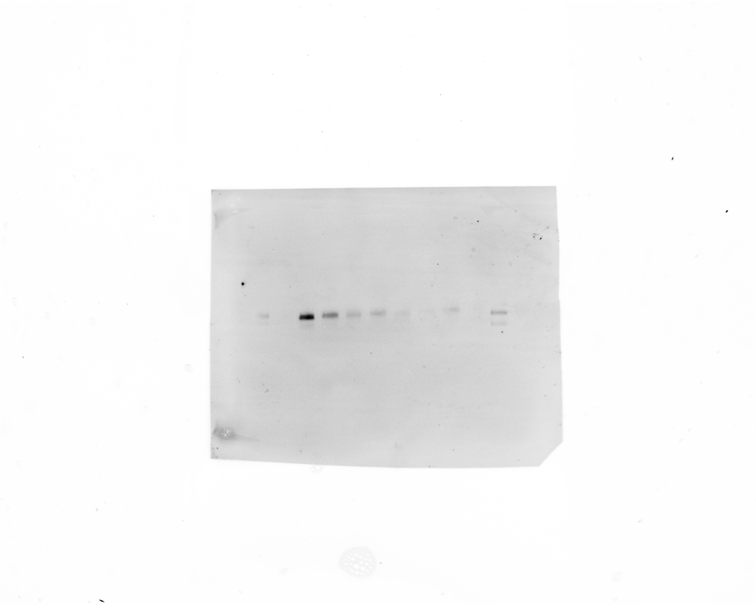

Full unedited gel for Figure 1B top right

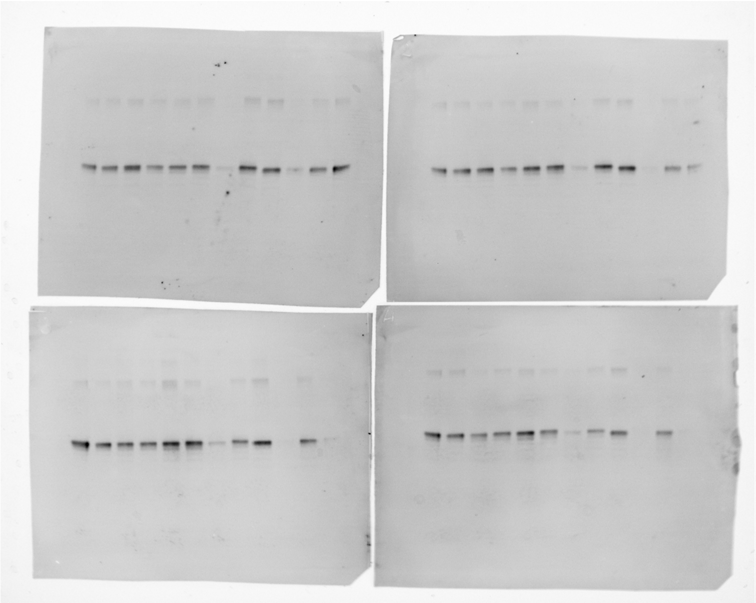

Full unedited gel for Figure 3A

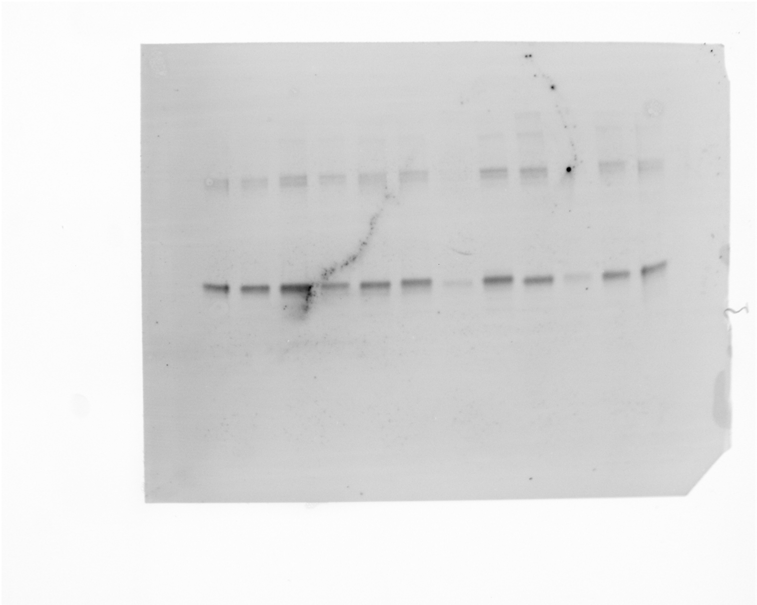

Full unedited gel for Figure 4A and 6A top (input)

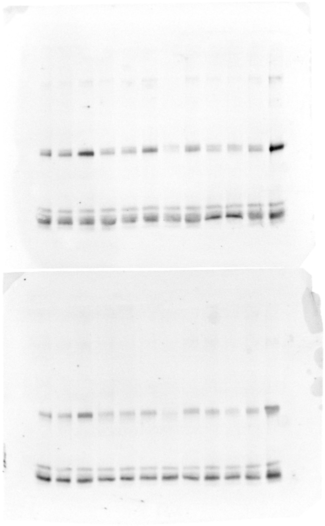

Full unedited gel for Figure 4A and 6A bottom (IP2c1 mAb and 8A7 mAb)

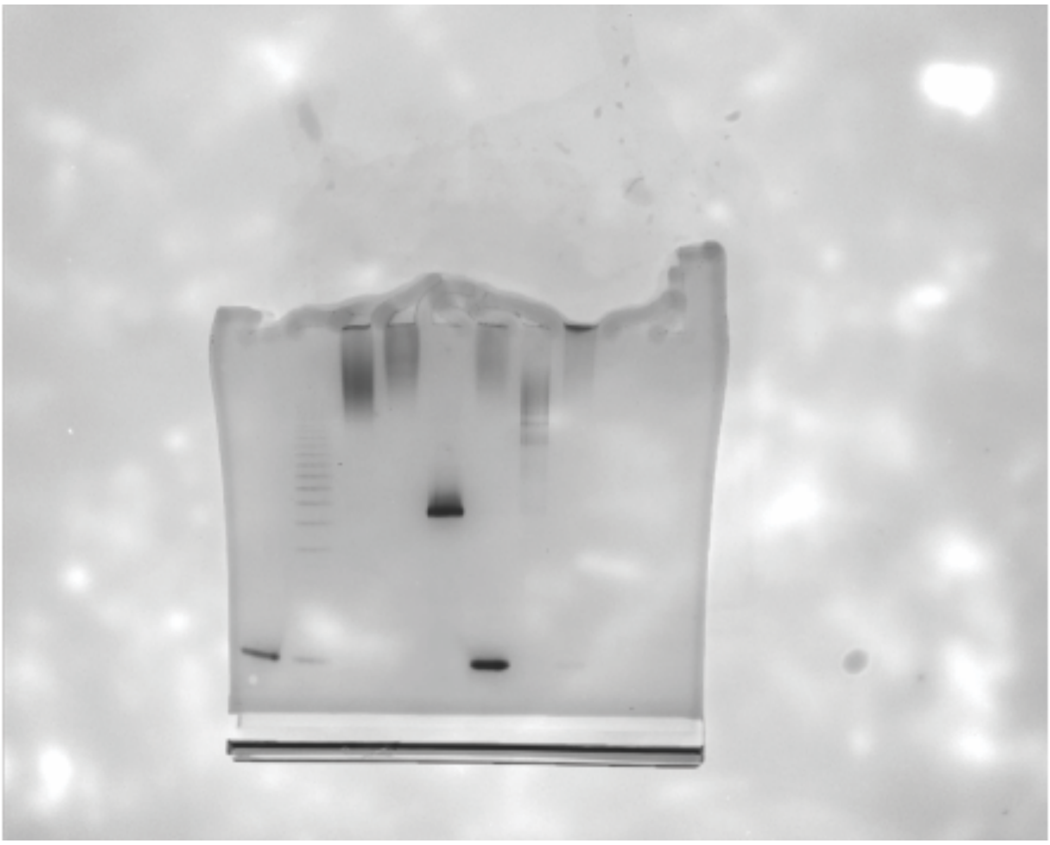

Full unedited gel for Figure 5D
